# Rare Copy Number Variants in Array-Based Comparative Genomic Hybridization in Early-Onset Skeletal Fragility

**DOI:** 10.3389/fendo.2018.00380

**Published:** 2018-07-10

**Authors:** Alice Costantini, Sini Skarp, Anders Kämpe, Riikka E. Mäkitie, Maria Pettersson, Minna Männikkö, Hong Jiao, Fulya Taylan, Anna Lindstrand, Outi Mäkitie

**Affiliations:** ^1^Department of Molecular Medicine and Surgery and Center for Molecular Medicine, Karolinska Institutet, Stockholm, Sweden; ^2^Center for Life Course Health Research, Faculty of Medicine, University of Oulu, Oulu, Finland; ^3^Faculty of Biochemistry and Molecular Medicine, University of Oulu, Oulu, Finland; ^4^Folkhälsan Institute of Genetics, University of Helsinki, Helsinki, Finland; ^5^Science for Life Laboratory, Department of Biosciences and Nutrition, Karolinska Institutet, Stockholm, Sweden; ^6^Department of Clinical Genetics, Karolinska University Hospital, Stockholm, Sweden; ^7^Children's Hospital, University of Helsinki and Helsinki University Hospital, Helsinki, Finland

**Keywords:** osteoporosis, bone fracture, cilia, copy number variant (CNV), array CGH

## Abstract

Early-onset osteoporosis is characterized by low bone mineral density (BMD) and fractures since childhood or young adulthood. Several monogenic forms have been identified but the contributing genes remain inadequately characterized. In search for novel variants and novel candidate loci, we screened a cohort of 70 young subjects with mild to severe skeletal fragility for rare copy-number variants (CNVs). Our study cohort included 15 subjects with primary osteoporosis before age 30 years and 55 subjects with a pathological fracture history and low or normal BMD before age 16 years. A custom-made high-resolution comparative genomic hybridization array with enriched probe density in >1,150 genes important for bone metabolism and ciliary function was used to search for CNVs. We identified altogether 14 rare CNVs. Seven intronic aberrations were classified as likely benign. Five CNVs of unknown clinical significance affected coding regions of genes not previously associated with skeletal fragility (*ETV1-DGKB, AGBL2, ATM, RPS6KL1-PGF*, and *SCN4A*). Finally, two CNVs were pathogenic and likely pathogenic, respectively: a 4 kb deletion involving exons 1–4 of *COL1A2* (NM_000089.3) and a 12.5 kb duplication of exon 3 in *PLS3* (NM_005032.6). Although both genes have been linked to monogenic forms of osteoporosis, *COL1A2* deletions are rare and *PLS3* duplications have not been described previously. Both CNVs were identified in subjects with significant osteoporosis and segregated with osteoporosis within the families. Our study expands the number of pathogenic CNVs in monogenic skeletal fragility and shows the validity of targeted CNV screening to potentially pinpoint novel candidate loci in early-onset osteoporosis.

## Introduction

Early-onset osteoporosis is characterized by low bone mineral density (BMD), compromised bone strength and increased susceptibility to fractures since childhood or young adulthood (1). Genetic variants rather than environmental factors are likely to play a key role in etiology (2, 3). Osteogenesis imperfecta (OI) is the most common form of early-onset primary osteoporosis (4). To date, mutations in around 17 genes with different inheritance patterns have been linked to OI and/or early-onset osteoporosis (5, 6). In addition, several other genes are known to cause skeletal syndromes featuring osteoporosis, such as spondylo-ocular syndrome (MIM 605822) caused by biallelic mutations in *XYLT2* (4, 7, 8). However, the genetic background of childhood skeletal fragility still remains inadequately explored.

Although the most frequent types of mutations underlying bone fragility are single nucleotide variants (SNVs) or small insertions or deletions of nucleotides (5), structural variants that rearrange the DNA on a larger scale have also been identified (9). These are often in the form of copy number variants (CNVs; deletions or duplications), where a certain DNA sequence is present in more or less copies than the reference genome (10). Many CNVs in the human genome represent benign normal variants but if the CNVs affect genes or regulatory regions not tolerating deletions or duplications they can give rise to genetic diseases.

Primary cilia, which are microtubule-based extensions present in most of the cell types in our body, are involved in the pathogenesis of several disorders, collectively named ciliopathies, which include chronic kidney disease, mental retardation, and skeletal dysplasia (11). Recently, cilia have emerged as important players in bone turnover and osteocytic mechanosensing (12–14). However, the potential link between cilia genes and primary osteoporosis remains unexplored.

To further elucidate the genetic background of early-onset skeletal fragility we carried out a cohort study assessing the spectrum of rare and pathogenic CNVs in a group of young subjects with osteoporosis and/or recurrent fractures. We used a custom-made high-resolution comparative-genomic hybridization (CGH) microarray with genome-wide coverage but increased probe density in the genes implicated in various skeletal diseases (>300) and in ciliary function (>850).

## Patients and methods

### Study cohorts

As part of an ongoing research program on genetic causes of early-onset osteoporosis we recruited 70 Finnish subjects to the present study. This study was carried out in accordance with the recommendations of Helsinki University Hospital Ethics Committee. The protocol was approved by the Institutional review board of Helsinki University Hospital. All subjects gave written informed consent in accordance with the Declaration of Helsinki.

The participants were enrolled as two separate cohorts using different inclusion criteria.

The first subgroup encompassed 15 unrelated patients [6 males (40%), median age at last follow-up = 22 years, range 7–55 years] with diagnosis of primary osteoporosis. Inclusion criteria for this group were: (1) BMD Z-score ≤−2.0, (2) at least three significant peripheral fractures and/or one or more spinal compression fracture(s), (3) no chronic illness leading to secondary osteoporosis, and (4) age ≤30 years at the time of osteoporosis diagnosis.

The second subgroup was more mildly affected than the first group and included 55 unrelated children [38 males (69%), median age at last follow-up 10 years, age range 6–16 years] who were enrolled during an epidemiological study on childhood fractures (15). All had sustained (1) at least two low-energy long bone fractures before 10 years of age or (2) three low-energy long bone fractures before age 16 years and/or (3) at least one low-energy vertebral compression fracture; BMD, which was low in some patients and normal in others, was not used as an inclusion criterion (16). All had undergone thorough pediatric evaluation with laboratory tests to exclude other illnesses that could lead to fractures and secondary osteoporosis, such as celiac disease, inflammatory bowel disease, hypogonadism, mineral disorders, or hypophosphatasia (16). One child also had attention deficit symptoms, one other child had mild learning difficulties and another child transient ocular symptoms (double vision and ptosis).

The control group included 67 healthy Finnish subjects (31 males, 46%) who served as controls in previous studies assessing genetic determinants of early-onset obesity (17, 18). Since the Finnish population differs genetically from others, we used this control group to exclude rare CNVs that were absent from the public Database of Genomic Variants (DGV) but present in healthy Finnish controls. The median age in the group was 20 years (range 15–25 years).

### Microarray-based comparative genomic hybridization (Array-CGH)

To identify novel pathogenic CNVs causing bone fragility we used high-resolution microarray-based comparative genomic hybridization (array-CGH). Our custom-designed array-CGH (design 2 × 400 k) had 180 k probes evenly distributed throughout the genome and an increased probe density (220 k probes) in over 300 genes causing skeletal diseases and over 850 genes involved in cilia proteome (Supplemental Table S1). Furthermore, our custom array, which is used in a wide range of research studies, also targeted genes involved in other conditions in addition to those possibly affecting bone metabolism (e.g., mental retardation). The average coverage in the specifically targeted genes was one oligonucleotideprobe (60 nucleotides in length) per 100 base pairs in the coding regions and one probe per 500 base pairs in non-coding regions (introns and 5′/3′ UTR). Slides were designed using the Agilent Technologies web portal eArray.

The experiments were performed according to standard procedures using 1.2 μg of genomic DNA. The DNA from each patient and each sex-matched control was digested with Alu I and Rsa I restriction enzymes (Sigma-Aldrich) and labeled using Enzo Life Sciences CGH Labeling kit for oligo arrays. The DNA samples were purified using the QIAquick PCR purification kit (Qiagen) and hybridized with the 2x Hi-RPM hybridization buffer (Agilent Technologies), Blocking Agent (Agilent Technologies), and Cot1 DNA (Invitrogen). The slides were washed with Wash Buffer 1-2 (Agilent Technologies) and acetonitrile (Sigma-Aldrich) and afterwards scanned on Agilent G2565CA Microarray Scanner. The files were extracted using Feature Extraction software version 10.7.3 and the results were analyzed using Agilent Genomic Workbench 7.0. The ADM-2 (aberration detection module) algorithm was used to calculate aberrations. To get automated calls and limit the number of false negatives we set up the following cut offs: (1) minimum of 4 consecutive probes, (2) minimum aberration size of 500 bp, (3) minimum absolute average log-ratio for amplifications >0.4, and (4) minimum absolute average log ratio for deletions >0.5. Regarding the array quality, most of the results we analyzed had an excellent Derivative Log Ratio Spread (DLRS) value (<0.20) and only few of them had a good DLRS (<0.25).

All identified aberrations were manually assessed and classified into three categories: benign, uncertain clinical significance, and pathogenic, according to the American College of Medical Genetics (ACMG) guidelines for CNVs (19). A CNV is classified as benign if it is reported in the Database of Genomic Variants (DGV) and/or present in healthy individuals and therefore not likely to cause an abnormal phenotype. The variants of uncertain clinical significance can be subdivided into three categories: (1) uncertain clinical significance; likely benign (2) uncertain clinical significance (no sub-classification); (3) uncertain clinical significance; likely pathogenic. In the following text, we only use the terms likely benign, uncertain clinical significance and likely pathogenic for convenience. All CNVs that are absent both from DGV and from our ethnically matched (Finnish) control group were considered as rare. Rare CNVs affecting non-coding regions are determined as likely benign. Rare CNVs located in coding regions of genes not yet characterized as causing abnormal bone phenotypes were defined as variants of uncertain clinical significance. Finally, novel CNVs in genes already linked to OI and/or early-onset osteoporosis were assigned as likely pathogenic. A CNV is classified as pathogenic according to the ACMG guidelines only if the exact CNV has already been reported in previous studies or if this CNV overlaps a smaller region that has already been shown to be of clinical relevance.

### Genetic validation of Array-CGH findings

Breakpoint PCR was used to pinpoint the breakpoints of the novel and pathogenic/likely pathogenic CNVs. Other family members were subsequently screened with the same method to establish if these CNVs were segregating with the disease. PCR reactions were performed using Platinum Taq polymerase. Subsequently, Sanger sequencing was performed according to standard procedures to sequence the breakpoints. Applied Biosystems 3730 DNA Analyzer and SeqScape (Applied Biosystems) were used, respectively, to sequence and analyze the results.

Whole-genome sequencing was also performed to validate a tandem duplication in one index subject and to exclude potential SNVs in other genes presently known to cause OI/osteoporosis. The libraries were prepared using the method Illumina TruSeq PCR-free (350 bp) method at Science for Life Laboratory (Stockholm) according to the manufacturer's instructions and sequenced on Illumina HiSeq X (Illumina, California, USA) to an average autosomal depth of 34.34X. In-house pipeline was used to generate and annotate variants according to best practice guidelines. The reads in the FASTQ files were aligned to the reference human genome (assembly GRCh37) by short read alignment program Burrows-Wheeler Aligner (20). Quality check of data and variant calling were performed using Genome Analysis Toolkit (GATK) (21) whereas variant annotation was carried out using Variant Effect Predictor (VEP) (22). The final data were filtered and analyzed with GEMINI (23). Integrative Genomics Viewer (IGV) was used to visualize the aligned reads and variants.

## Results

Overall, we identified 14 rare CNVs (Table 1) in 12 out of 70 patients (two patients had 2 rare CNVs, one of uncertain clinical significance and one likely benign). All the CNVs were identified in male patients in a heterozygous or hemizygous state.

**Table 1:** Rare CNVs identified in 12 patients with skeletal fragility.

**chr**	Start^*^	Stop^*^	**Size (bp)**	**CNV**	**# Probes**	**Gene**	**Location**	**Outcome**	**Patient's gender**	**Clinical information**
1	212761003	212762859	1856	del	4	*ATF3*	Intronic	Likely benign	M	Susceptibility to fractures
1	237592436	237598912	6476	del	11	*RYR2*	Intronic	Likely benign	M	Susceptibility to fractures
2	214386320	214409233	22913	del	31	*SPAG16*	Intronic	Likely benign	M	Susceptibility to fractures
3	11440479	11462041	21562	del	16	*ATG7*	Intronic	Likely benign	M	Susceptibility to fractures
5	11439519	11443819	4300	del	7	*CTNND2*	Intronic	Likely benign	M ^(A)^	Susceptibility to fractures
7	108058010	108069358	11348	del	17	*NRCAM*	Intronic	Likely benign	M ^(B)^	Susceptibility to fractures
7	147887539	147898084	10545	del	15	*CNTNAP2*	Intronic	Likely benign	M	Susceptibility to fractures
7	12878014	14503169	1625155	del	52	*ETV1, DGKB*	Exonic	Uncertain clinical significance	M ^(B)^	Susceptibility to fractures
11	47722207	47728816	6609	del	13	*AGBL2*	Exonic	Uncertain clinical significance	M	Susceptibility to fractures
11	108233815	108240487	6672	dup	34	*ATM*	Exonic	Uncertain clinical significance	M	Susceptibility to fractures
14	75383413	75465561	82148	del	5	*RPS6KL1, PGF*	Exonic	uncertain clinical significance	M	Susceptibility to fractures
17	62025315	62026930	1615	del	6	*SCN4A*	Exonic	Uncertain clinical significance	M ^(A)^	Susceptibility to fractures
7	94024366	94028364	3998	del	9	*COL1A2*	Exonic	Pathogenic	M	Primary osteoporosis
X	114848591	114859994	11403	dup	28	*PLS3*	Exonic	Likely pathogenic	M	Primary osteoporosis

* *Coordinates given in GRCh37; chr, chromosome; del, deletion; dup, duplication (A) and (B) indicates, respectively, the two patients both having two rare CNVs*.

Half of the identified variants (*n* = 7) were located in intronic regions of protein-coding genes. Three CNVs were identified in genes that have been associated with dyslexia (*CTNND2, NRCAM*, and *CNTNAP2*) and their role in bone is currently unknown (24–26). One deletion was identified in *ATG7*, encoding Autophagy Related 7, an essential protein for autophagy (27). Finally, three other deletions were found respectively in *SPAG16*, playing a role in the axoneme of the sperm, *RYR2*, whose mutations have been associated to cardiac diseases and *ATF3*, a candidate gene for hypospadias (28–30). Most of these CNVs targeted genes that were included in our custom design to screen patients affected by other diseases than those related to bone or ciliopathies. Moreover, all these intronic variants locate far from the splicing acceptor/donor sites and thus the possibility of affecting the splicing mechanism is rather unlikely. Finally, intronic CNVs are generally neutral and although it is not possible to exclude the activation of cryptic splice sites we considered these CNVs as likely benign.

Seven other rare CNVs affected coding regions of nine genes (*ETV1, DGKB, AGBL2, ATM, RPS6KL1, PGF, SCN4A, COL1A2*, and *PLS3*) in total (Table 1). Five of these rare CNVs were classified as variants of uncertain clinical significance since the involved genes have not previously been linked to skeletal fragility (Table 2). One variant duplicates the last part of *ATM* starting from intron 62/63 (NM_000051.3). The other variants were deletions: (1) an intragenic deletion (intron 14—exon 17) in *SCN4A* (NM_000334.4) (2) a ~1.6 Mb deletion of the entire *ETV1* and exons 21-25 of *DGKB* (NM_004080.2) (3) a deletion of exons 5-7 in *AGBL2* (NM_024783.3) and (4) a deletion of exons 1-4 in *RPS6KL1* (NM_031464.4) and the entire *PGF* gene. *AGBL2* was included in our design because it plays a role in cilia. The other CNVs were instead detected because our array design also targets genes related to other phenotypes than those related to bone or because they were large enough to be captured by our backbone coverage. Among these CNVs, the deletions affecting *SCN4A*, and *ETV1* could potentially contribute to bone fragility in our patients since these genes are related to bone or muscles (Table 2).

**Table 2:** Proteins encoded by the genes involved in the 5 variants of uncertain significance and clinical information for the affected patients.

**Gene**	**Protein**	**Protein function**	**Patient's Age**	**LS BMD Z-score**	**No. of compression fractures**	**No. of long bone fractures**
*ATM*	ATM Serine/Threonine Kinase	Cell cycle checkpoint kinase. Disease: Ataxia-telangiectasia	10	0	0	2
*SCN4A*	Sodium Voltage-Gated Channel Alpha Subunit 4	Generation and propagation of action potentials in neurons and muscle. Disease: Myotonia congenita	15	−1.8	0	3
*ETV1, DGKB*	ETS Variant 1	Cell growth, angiogenesis, migration, proliferation and differentiation. Diseases: Ewing sarcoma (*ETV1*-*EWS* translocations). Prostate cancer (*ETV1-TMPRSS2* translocations)	13	−1.7	1	1
	Diacylglycerol Kinase Beta	Unknown				
*AGBL2*	ATP/GTP Binding Protein Like 2	Mediation of deglutamylation of target proteins	7	−0.8	1	3
*RPS6KL1, PGF*	Ribosomal Protein S6 Kinase Like 1	Unknown	12	+0.5	0	4
	Placental Growth Factor	Angiogenesis and endothelial cell growth. VEGF signaling pathway				

**Figure 1:**
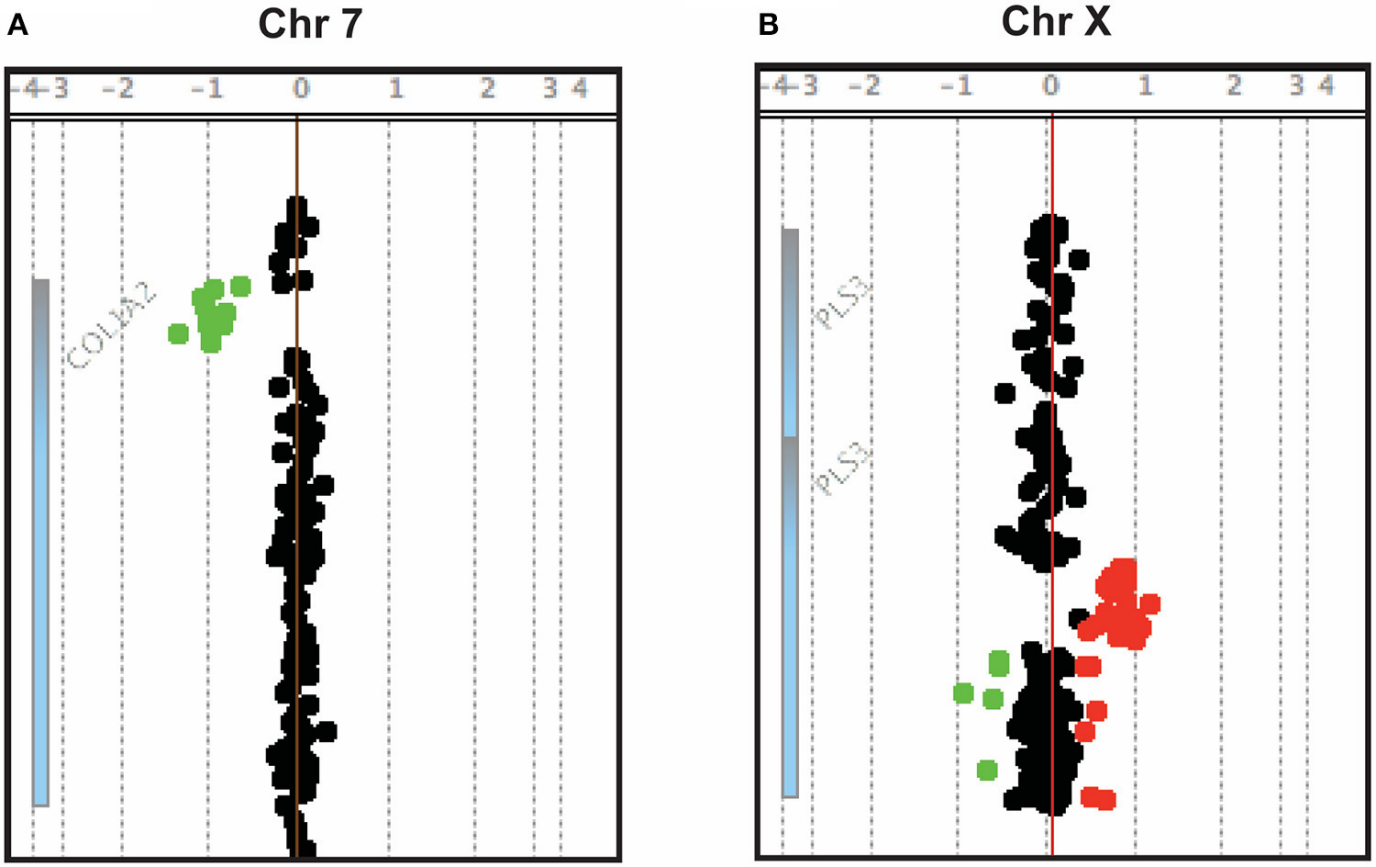
Array-CGH snapshots showing a ~4 kb deletion in *COL1A2* **(A)** and a ~12.5 kb duplication in *PLS3* **(B)**.

**Figure 2:**
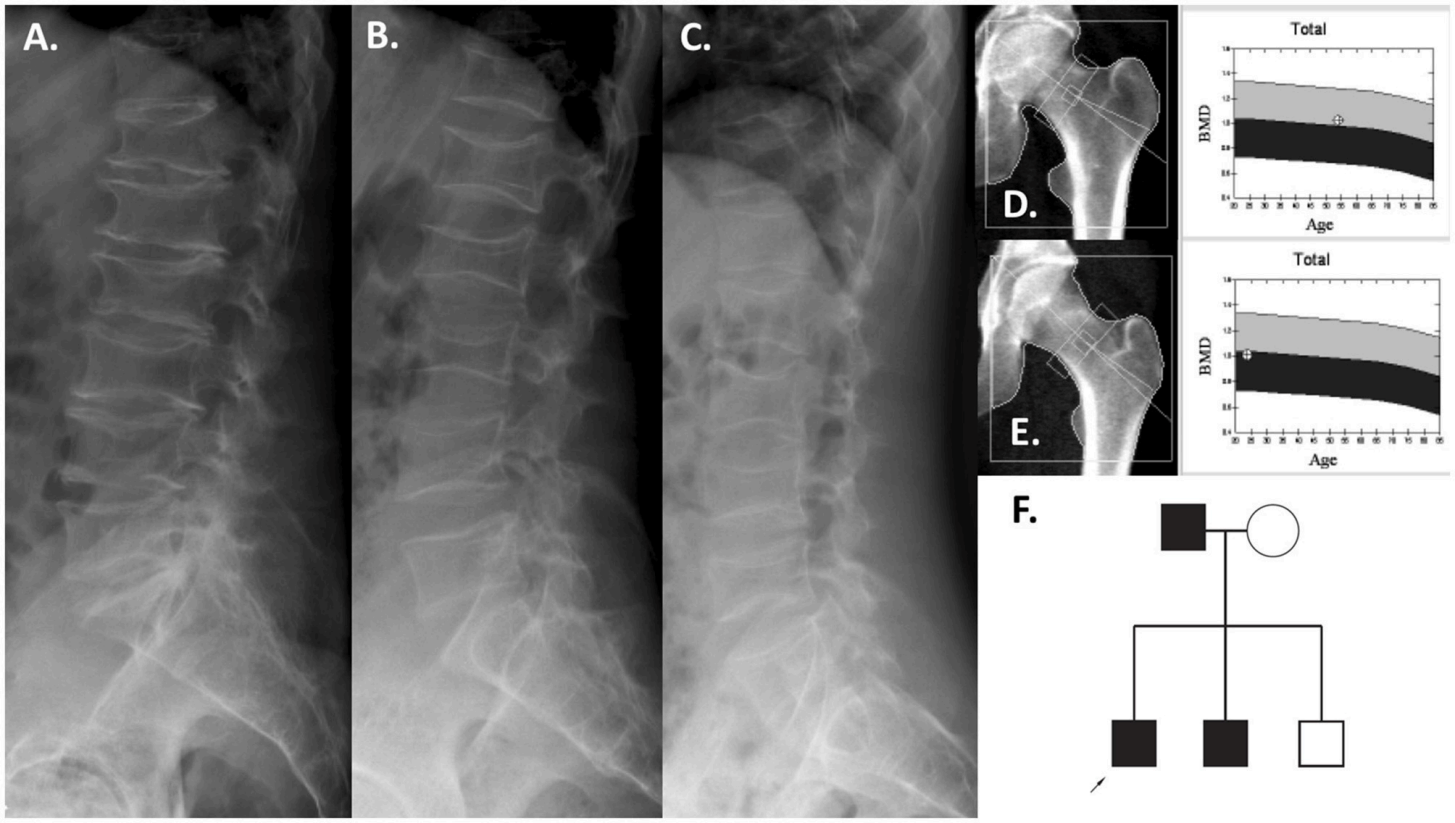
Patients with the *COL1A2* deletion. Lumbar spine radiographs of the father at 54 years **(A)**, the index patient at 24 years **(B)**, and brother at 21 years **(C)**. The proximal hip BMD was normal (father, **D**; index patient, **E**). Pedigree of the family **(F)**.

Two CNVs were regarded as pathogenic and likely pathogenic, respectively, as they affected coding regions of genes already known to cause early-onset skeletal fragility (*COL1A2* and *PLS3*; Table 1). They were identified in two patients with primary osteoporosis. The first CNV is a novel ~4 kb heterozygous deletion, chr7: 94024366- 94028364 (reference genome: GRCh37), affecting the *COL1A2* gene (Figure 1A). *COL1A2* encodes the α2 chain of type I collagen and mutations give rise to different bone diseases, including autosomal dominant OI and Ehlers-Danlos syndrome. The breakpoints of the deletion were determined to be in exon 1 and intron 4 of *COL1A2* (NM_000089.3; Supplemental Figure S1); the deletion is thus predicted to remove the amino acids 8–14 of the N-propeptide in the immature protein and lead to a frameshift that introduces an early-stop codon in the protein [g.491_5060del (p.R8Ffs^*^14)]. In this way this CNV was treated as a frameshift SNV and classified as pathogenic according to the ACMG classification of sequence variants (31). The affected index patient is a 36-years-old man with severe early-onset osteoporosis, low BMD and several compression fractures since the age of 8 years (Figure 2B). Previous measurements of metabolic bone markers, including serum alkaline phosphatase and aminoterminal propeptide of type I collagen as well as urinary collagen type 1 cross-linked N-telopeptide, were all within normal limits. Subsequent segregation analysis identified the same CNV in the index patient's affected father (66 years old) and affected brother (34 years old), both presenting with a similar phenotype (Figure 2F). The probability of co-segregation of the disease in multiple family members was evaluated using The American College of Medical Genetics and Genomics and Association of Molecular Pathology (ACMG-AMP) evidence level (32). Assuming complete penetrance, a single causal allele and no phenocopies the formula is *N* = 1/BF, as BF is defined by the Thompson-Bayrak-Toydemir BF method (33). Under these considerations the denominator BF is calculated as (1/2)^m^ where “m” is the number of meiosis of the variant of interest. In our case *N* = 1/8 since we observed 3 meiosis supporting the co-segregation (in the two affected brothers and in the healthy brother) while the threshold for “pathogenic supporting” evidence level is 1/8 for single families.

Interestingly, the severe vertebral compression fractures identified in the index patient (Figure 2B) were almost identical in his father (Figure 2A) and his affected brother (Figure 2C), affecting the entire spine. Despite these severe spinal changes, the proximal femur BMD was normal in all (Figures 2D,E). All three affected individuals lacked the typical OI features such as blue sclerae, joint laxity, or dentinogenesis imperfecta.

**Figure 3:**
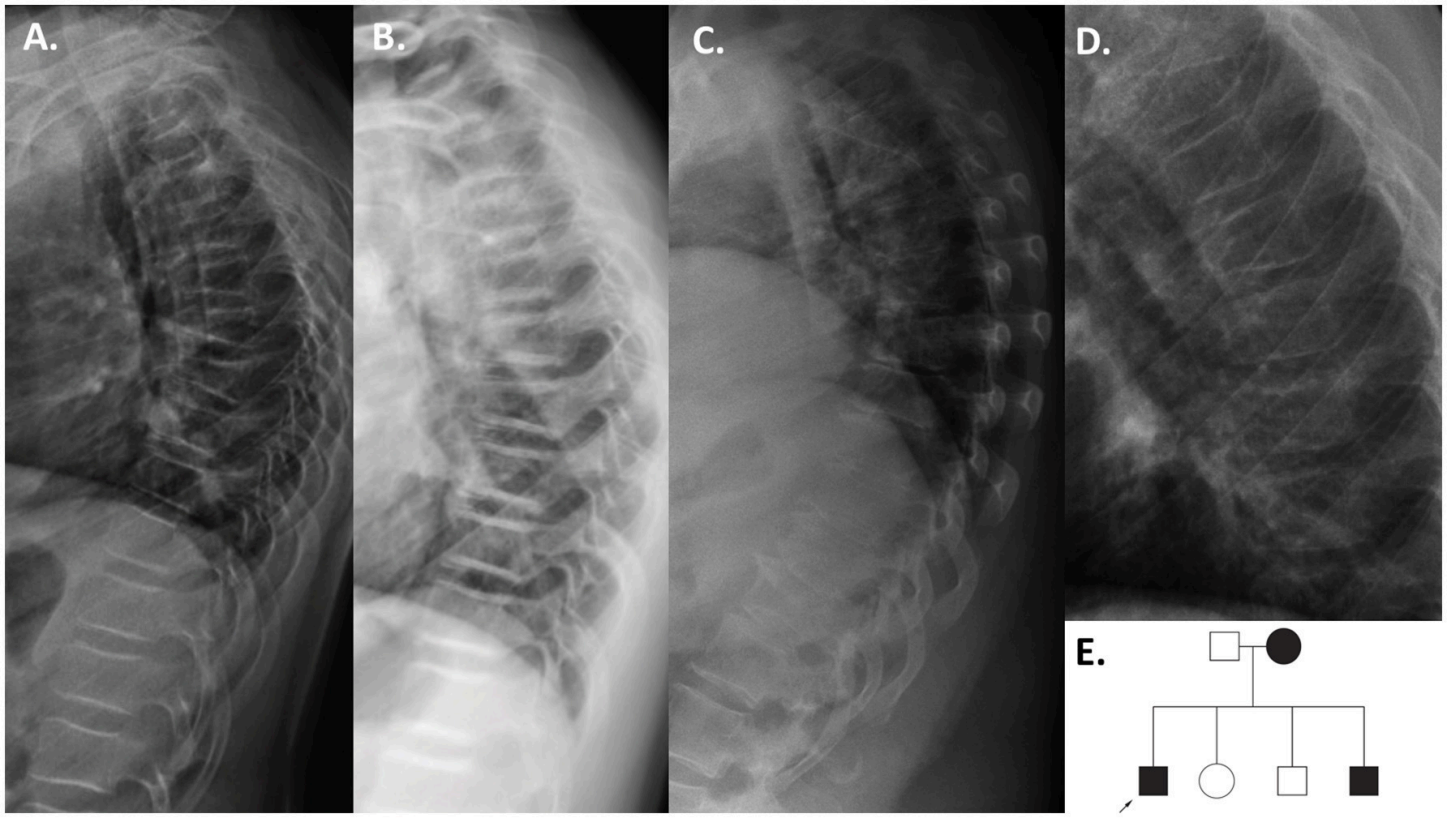
Patients with the *PLS3* duplication. Thoracic radiographs of the index patient before bisphosphonate treatment at age 11 years **(A)**, and after 2 years of bisphosphonate treatment at 13 years **(B)** showing multiple compressed vertebrae and improvement in vertebral shape after bisphosphonate treatment. The mutation-positive mother at 40 years **(C)** and younger brother at 6 years **(D)** also had compression fractures. Pedigree of the family **(E)**.

The second finding identified by array-CGH is a novel ~12.5 kb duplication, chrX: 114,848,381–114,860,880 (reference genome: GRCh37), within *PLS3* (Figure 1B). *PLS3* encodes plastin 3 and mutations in this gene underlie X-linked osteoporosis. The aberration starts in intron 2 and ends in intron 3 of *PLS3* (NM_005032.6). Breakpoint PCR showed that the duplication is in tandem. The change was identified in a 21-year-old male affected by severe osteoporosis. He has sustained 10 metatarsal fractures and spinal compression fractures since childhood (Figure 3A) and has low BMD values (lumbar spine Z-score−3.1). The aforementioned bone turnover markers were all normal. He was treated with bisphosphonates from the age of 11 to 13 years with a good response (Figure 3B). By investigating other family members, we identified the same duplication in a 7-year-old brother and their mother (Figure 3E), both of them affected by skeletal symptoms. The variant was instead absent in the two healthy siblings. In this case the ACMG-AMP evidence level for classifying the variant based on the co-segregation score was achieved (*N* = 1/16) and the variant was thus defined as pathogenic according to this probabilistic measure. His mother had low BMD, back pain, and spinal compression fractures (Figure 3C) and his younger brother had bone pain, low BMD, 3 previous long bone fractures and vertebral fractures (Figure 3D). Since the duplication is in tandem it might affect the splicing mechanism and/or lead to a frameshift in the reading frame resulting in a premature termination codon on mRNA level. The finding was confirmed by whole genome sequencing (WGS) (Supplemental Figure S2). The analysis of the WGS data did not detect any candidate variant other than the CNV in *PLS3* that could explain the disease in the patient.

## Discussion

Our study aimed to identify novel rare and pathogenic CNVs in a group of young Finnish patients with mild to severe bone fragility. Our study identified several rare CNVs. Altogether there were two exonic pathogenic/likely pathogenic variants in 2 out of 70 patients (3%). These CNVs were identified in two index patients with primary osteoporosis and the affected genes were already linked to this disease. The first partially deletes part of the N-terminal signal peptide *COL1A2*, g.491_5060del, and is predicted to cause a frameshift at Arginine 8 that ends with an early stop codon at Cysteine 22 (p.R8Ffs^*^14). The signal peptide determines the secretion efficiency of collagen pre-pro-peptide into the endoplasmic reticulum. It is known that the range of OI severity depends on the location of mutation in type I collagen. Mutations in the Gly-X-Y repeats of the helical region and in the C-propeptide cause mild to severe OI (5, 6). Furthermore, mutations in the N-propeptidase cleavage site give rise to the Ehlers Danlos syndrome VIIA (if the mutation affects *COL1A1*) or VIIB (if the mutation resides in *COL1A2*) (34) and mutations in the C- propeptidase cleavage site cause high-bone mass OI (35). Finally, a particular form of combined OI and Ehlers Danlos syndrome derives from heterozygous mutations in the N-propeptide (36).

To date, only few deletions in *COL1A2* have been reported and the exact deletion identified in our patient and in some of his family members has never been described. Previous studies showed that multi-exonic *COL1A2* deletions preserving the Gly-X-Y pattern may anyway lead to defective folding of type I collagen (34, 37, 38). Furthermore, deletions in the other type I collagen encoding gene, *COL1A1*, give rise to different OI phenotypes of variable severity. In general, complete *COL1A1* deletions cause milder phenotypes compared to multi-exon deletions (9). Haploinsufficiency, due to a reduced COL1A2 mRNA expression, may explain the severity of the bone disease in our patient. Since the deletion occurs in the first amino acids, it is likely that the mRNA produced by the affected allele is degraded by nonsense-mediated RNA decay. In this way, there would be no effective protein synthesis from this allele. Our patient and his affected family members all presented with about identical spinal fractures involving almost all vertebral bodies and lacked the typical features of OI or Ehlers Danlos syndrome. The special location of the deletion probably explains why the patient's phenotype was not typical for OI.

Concerning the second CNV, the duplication within *PLS3*, chrX: 114,848,381–114,860,880, is the first duplication described in this gene. On the other hand, SNVs and deletions in *PLS3* have already been reported in patients with osteoporosis (39–43). Recently, some patients with *PLS3* deletions have been described, and apart from severe osteoporosis there is a bone mineralization defect (42). However, the molecular mechanism behind PLS3 osteoporosis is not fully understood yet. Concerning the identified duplication, it affects the EF-hand 1 domain, which is very important for calcium binding. This tandem duplication is likely to cause a defective splicing and/or a frameshift that leads to an early-stop codon in the mRNA. Since *PLS3* locates on the X chromosome the index patient and his affected brother are likely to not have any functional protein, which is concordant with their severe skeletal phenotype. On the other hand their mother, who has also a wild-type copy of the gene, is only mildly affected.

Half of the other 10 rare CNVs were regarded as being of unknown clinical significance and half as benign. Among the variants of unknown clinical significance, one deletion was identified in *AGBL2*, encoding the ATP/GTP binding protein like 2, a protein whose function is not well understood yet. Agbl2/Agbl3 double-KO mice present with defects in tubulin deglutamylation in testis and sperm but no skeletal fragility (44). Furthermore, the duplication in *ATM* was also regarded as likely benign since mutations in this gene have been seen in patients with ataxia talangiectasia (45). However, three variants could potentially have an impact on the skeleton. While our patient with a deletion within *SCN4A* lacked symptoms of myotonia congenita, it is possible that the deletion leads to mild muscular symptoms and thereby to reduced bone strength (46). The second potential deleterious deletion affects *RPS6KL1* and *PGF*. Although *PGF* does not seem to be involved in bone metabolism, *RPS6KL1* might affect BMD (47). In fact, one of the largest genome-wide association studies (GWAS) on BMD showed a hit in *RPS6KA5*, which belongs to the same family of ribosomal protein kinases as *RPS6KL1* (48). The third variant that could be the cause of mild skeletal fragility affects *ETV1* and *DGKB*. Somatic translocations of *ETV1* have indeed been found not only in prostate cancer but also in Ewing sarcoma (49, 50). *DGKB* defects have been linked to glucose homeostasis but to the best of our knowledge there are no studies or mouse models showing abnormal bone phenotypes due to mutations in this gene (51). In summary, although we did not have enough evidence to show that these rare exonic CNVs cause skeletal fragility, they could be regarded as new potential loci for osteoporosis and should be further investigated.

Interestingly, 3 out of 7 intronic CNVs affected genes associated with neuronal impairment (*CTNND2, NRCAM*, and *CNTNAP2*). A potential effect on bone metabolism could either be direct, leading to altered bone cell function by impairing e.g., osteocytic cilia function, or indirect, affecting the neuronal factors involved in skeletal regulation. Previous studies in mice have shown that neurons could also influence BMD, in addition to the central nervous system (52, 53). The patient with multiple fractures and the CNV in *CTNND2* had transient problems in vision whereas the patient with the CNV in *CNTNAP2* had mild attention deficit symptoms. It is also possible that the high number of bone fractures could result from a reduced capacity of our patients to adjust reflexes during falls, leading to propensity to fractures. However, due to the intronic location of these variants and absence of functional validation, their relationship to skeletal fragility in our patients remains uncertain.

A limitation of our study is that we only had increased coverage in >1,150 genes that were already known to be somehow related to bone homeostasis or ciliary function (plus other loci included in our custom design for other research purposes). For this reason, this method is valid for identification of novel candidate genes outside these only when large deletions or duplications occur (e.g., *RPS6KL1-PGF* and *ETV1-DGKB* deletions). Despite this limitation, we identified several potentially interesting CNVs that need to be explored in future studies.

In conclusion, we showed the validity of our custom-made array-CGH to expand the spectrum of large-scale variants in skeletal fragility by identifying a novel multi-exonic deletion in the *COL1A2* gene and a novel duplication of the entire exon 3 of *PLS3*. Finally, we showed a potential use of our array-CGH to also target cilia genes and possibly identify novel candidate loci for early-onset skeletal fragility. However, the significance of rare CNVs in genes not yet linked to skeletal phenotypes has to be further investigated.

## Author contributions

AC, AL, and OM: study design; AC and SS: study conduct and data analysis; AC, AL, SS, AK, RM, MP, FT, HJ, MM, and OM: data interpretation; AC, RM, and OM: drafting manuscript. All authors: data collection, revising manuscript content, and approving final version of manuscript. AC and OM take responsibility for the integrity of the data analysis.

### Conflict of interest statement

The authors declare that the research was conducted in the absence of any commercial or financial relationships that could be construed as a potential conflict of interest.

## Supplementary material

Supplemental Table S1 List of skeletal/cilia genes included in our custom-made array-CGH design.

Supplemental Figure S1 Validation of the pathogenic finding in *COL1A2* by breakpoint PCR followed by Sanger sequencing. The deletion starts in exon 1 and ends in intron 4.

Supplemental Figure S2 Validation of the likely pathogenic finding in *PLS3* by WGS. An increased coverage and a discordant orientation of some paired-end reads (red lines on the top of the figure) indicates the presence of a tandem duplication starting in intron 2 and ending in intron 3 of *PLS3* (coordinates chrX: 114,848,500–114,860,900). On the right part of the figure a zoomed-in snapshot represents the area delimited by the green rectangle. The discordant pairs in this region are due to the misalignment of several reads (colorful reads) caused by the presence of a highly repetitive region (long red stretch on the bar below these reads).

## References

[1] Van Den Bergh JP Van Geel TA Geusens PP. Osteoporosis, frailty and fracture: implications for case finding and therapy. Nat Rev Rheumatol. (2012) 8:163–72. 10.1038/nrrheum.2011.217 22249162

[2] Makitie O. Causes, mechanisms and management of paediatric osteoporosis. Nat Rev Rheumatol. (2013) 9:465–75. 10.1038/nrrheum.2013.45 23591487

[3] Rivadeneira F Makitie O. Osteoporosis and bone mass disorders: from gene pathways to treatments. Trends Endocrinol Metab. (2016) 27:262–81. 10.1016/j.tem.2016.03.006 27079517

[4] Van Dijk FS Sillence DO. Osteogenesis imperfecta: clinical diagnosis, nomenclature and severity assessment. Am J Med Genet A (2014) 164A:1470–81. 10.1002/ajmg.a.36545 24715559 PMC4314691

[5] Forlino A Marini JC. Osteogenesis imperfecta. Lancet (2016) 387:1657–71. 10.1016/S0140-6736(15)00728-X 26542481 PMC7384887

[6] Marini JC Forlino A Bachinger HP Bishop NJ Byers PH Paepe A Osteogenesis imperfecta. Nat Rev Dis Primers (2017) 3:17052. 10.1038/nrdp.2017.52 28820180

[7] Bonafe L Cormier-Daire V Hall C Lachman R Mortier G Mundlos S. Nosology and classification of genetic skeletal disorders: 2015 revision. Am J Med Genet A (2015) 167A:2869–92. 10.1002/ajmg.a.37365 26394607

[8] Costantini A Makitie O. Value of rare low bone mass diseases for osteoporosis genetics. Bonekey Rep (2016) 5:773. 10.1038/bonekey.2015.143 26793304 PMC4704609

[9] Bardai G Lemyre E Moffatt P Palomo T Glorieux FH Tung J. Osteogenesis imperfecta type I caused by COL1A1 deletions. Calcif Tissue Int (2016) 98:76–84. 10.1007/s00223-015-0066-6 26478226

[10] Zarrei M MacDonald JR Merico D Scherer SW. A copy number variation map of the human genome. Nat Rev Genet. (2015) 16:172–83. 10.1038/nrg3871 25645873

[11] Vertii A Bright A Delaval B Hehnly H Doxsey S. New frontiers: discovering cilia-independent functions of cilia proteins. EMBO Rep. (2015) 16:1275–87. 10.15252/embr.201540632 26358956 PMC4766453

[12] Nguyen AM Jacobs CR. Emerging role of primary cilia as mechanosensors in osteocytes. Bone (2013) 54:196–204. 10.1016/j.bone.2012.11.016 23201223 PMC3624072

[13] Schou KB Pedersen LB Christensen ST. Ins and outs of GPCR signaling in primary cilia. EMBO Rep. (2015) 16:1099–113. 10.15252/embr.201540530 26297609 PMC4576980

[14] Johnson CA Collis SJ. Ciliogenesis and the DNA damage response: a stressful relationship. Cilia (2016) 5:19. 10.1186/s13630-016-0040-6 27335639 PMC4916530

[15] Mayranpaa MK Makitie O Kallio PE. Decreasing incidence and changing pattern of childhood fractures: a population-based study. J Bone Miner Res. (2010) 25:2752–9. 10.1002/jbmr.155 20564246

[16] Mayranpaa MK Viljakainen HT Toiviainen-Salo S Kallio PE Makitie O. Impaired bone health and asymptomatic vertebral compressions in fracture-prone children: a case-control study. J Bone Miner Res. (2012) 27:1413–24. 10.1002/jbmr.1579 22367922

[17] Viljakainen H Andersson-Assarsson JC Armenio M Pekkinen M Pettersson M Valta H. Low copy number of the AMY1 locus is associated with early-onset female obesity in Finland. PLoS ONE (2015) 10:e0131883. 10.1371/journal.pone.0131883 26132294 PMC4489572

[18] Pettersson M Viljakainen H Loid P Mustila T Pekkinen M Armenio M. Copy number variants are enriched in individuals with early-onset obesity and highlight novel pathogenic pathways. J Clin Endocrinol Metab. (2017) 102:3029–39. 10.1210/jc.2017-00565 28605459

[19] Kearney HM Thorland EC Brown KK Quintero-Rivera F South ST Working Group of the American College of Medical Genetics Laboratory Quality Assurance C. American College of Medical Genetics standards and guidelines for interpretation and reporting of postnatal constitutional copy number variants. Genet Med. (2011) 13:680–5. 10.1097/GIM.0b013e3182217a3a 21681106

[20] Li H Durbin R. Fast and accurate short read alignment with Burrows-Wheeler transform. Bioinformatics (2009) 25:1754–60. 10.1093/bioinformatics/btp324 19451168 PMC2705234

[21] Van Der Auwera GA Carneiro MO Hartl C Poplin R Del Angel G Levy-Moonshine A From FastQ data to high confidence variant calls: the Genome Analysis Toolkit best practices pipeline. Curr Protoc Bioinformatics (2013) 43.11.10:11–33. 10.1002/0471250953.bi1110s43 PMC4243306 25431634

[22] McLaren W Gil L Hunt SE Riat HS Ritchie GR Thormann A. The ensembl variant effect predictor. Genome Biol (2016) 17:122. 10.1186/s13059-016-0974-4 27268795 PMC4893825

[23] Paila U Chapman BA Kirchner R Quinlan AR. GEMINI: integrative exploration of genetic variation and genome annotations. PLoS Comput Biol. (2013) 9:e1003153. 10.1371/journal.pcbi.1003153 23874191 PMC3715403

[24] Docherty SJ Davis OSP Kovas Y Meaburn EL Dale PS Petrill SA. A genome-wide association study identifies multiple loci associated with mathematics ability and disability. Genes Brain Behav. (2010) 9:234–47. 10.1111/j.1601-183X.2009.00553.x 20039944 PMC2855870

[25] Rodenas-Cuadrado P Ho J Vernes SC. Shining a light on CNTNAP2: complex functions to complex disorders. Eur J Hum Genet. (2014) 22:171–8. 10.1038/ejhg.2013.100 23714751 PMC3895625

[26] Hofmeister W Nilsson D Topa A Anderlid BM Darki F Matsson H. CTNND2-a candidate gene for reading problems and mild intellectual disability. J Med Genet. (2015) 52:111–22. 10.1136/jmedgenet-2014-102757 25473103

[27] Komatsu M Wang QJ Holstein GR Friedrich VL Jr Iwata J Kominami E. Essential role for autophagy protein Atg7 in the maintenance of axonal homeostasis and the prevention of axonal degeneration. Proc Natl Acad Sci USA. (2007) 104:14489–94. 10.1073/pnas.0701311104 17726112 PMC1964831

[28] Beleza-Meireles A Tohonen V Soderhall C Schwentner C Radmayr C Kockum I. Activating transcription factor 3: a hormone responsive gene in the etiology of hypospadias. Eur J Endocrinol. (2008) 158:729–39. 10.1530/EJE-07-0793 18426833

[29] Nagarkatti-Gude DR Jaimez R Henderson SC Teves ME Zhang ZB Strauss JF. Spag16, an axonemal central apparatus gene, encodes a male germ cell nuclear speckle protein that regulates SPAG16 mRNA expression. PLoS ONE (2011) 6:e20625. 10.1371/journal.pone.0020625 21655194 PMC3105110

[30] Dobrev D Wehrens XH. Role of RyR2 phosphorylation in heart failure and arrhythmias: controversies around ryanodine receptor phosphorylation in cardiac disease. Circ Res. (2014) 114:1311–9; discussion: 1319. 10.1161/CIRCRESAHA.114.300568 24723656 PMC4008932

[31] Richards S Aziz N Bale S Bick D Das S Gastier-Foster J. Standards and guidelines for the interpretation of sequence variants: a joint consensus recommendation of the American College of Medical Genetics and Genomics and the Association for Molecular Pathology. Genet Med. (2015) 17:405–24. 10.1038/gim.2015.30 25741868 PMC4544753

[32] Jarvik GP Browning BL. Consideration of cosegregation in the pathogenicity classification of genomic variants. Am J Hum Genet. (2016) 98:1077–81. 10.1016/j.ajhg.2016.04.003 27236918 PMC4908147

[33] Thompson D Easton DF Goldgar DE. A full-likelihood method for the evaluation of causality of sequence variants from family data. Am J Hum Genet. (2003) 73:652–5. 10.1086/378100 12900794 PMC1180690

[34] Schwarze U Hata R McKusick VA Shinkai H Hoyme HE Pyeritz RE. Rare autosomal recessive cardiac valvular form of Ehlers-Danlos syndrome results from mutations in the COL1A2 gene that activate the nonsense-mediated RNA decay pathway. Am J Hum Genet. (2004) 74:917–30. 10.1086/420794 15077201 PMC1181985

[35] Lindahl K Barnes AM Fratzl-Zelman N Whyte MP Hefferan TE Makareeva E. COL1 C-propeptide cleavage site mutations cause high bone mass osteogenesis imperfecta. Hum Mutat. (2011) 32:598–609. 10.1002/humu.21475 21344539 PMC3103631

[36] Cabral WA Makareeva E Colige A Letocha AD Ty JM Yeowell HN. Mutations near amino end of alpha1(I) collagen cause combined osteogenesis imperfecta/Ehlers-Danlos syndrome by interference with N-propeptide processing. J Biol Chem. (2005) 280:19259–69. 10.1074/jbc.M414698200 15728585

[37] Chessler S Byers P Defective folding and stable association with protein disulfide isomerase/prolyl hydroxylase of type I procollagen with a deletion in the ProaZ(1) chain that preserves theG ly-X-Y repeat pattern. J Biol Chem. (1992) 267:7751–7. 1339453

[38] Mundlos S Chan D Weng Y Sillence D Cole W Bateman J. Multiexon deletions in the type I collagen COL1A2 gene in osteogenesis imperfecta Type IB. J Biol Chem. (1996) 271:21068–74. 10.1074/jbc.271.35.21068 8702873

[39] Van Dijk FS Zillikens MC Micha D Riessland M Marcelis CL De Die-Smulders CE. PLS3 mutations in X-linked osteoporosis with fractures. N Engl J Med. (2013) 369:1529–36. 10.1056/NEJMoa1308223 24088043

[40] Fahiminiya S Majewski J Al-Jallad H Moffatt P Mort J Glorieux FH. Osteoporosis caused by mutations in PLS3: clinical and bone tissue characteristics. J Bone Miner Res. (2014) 29:1805–14. 10.1002/jbmr.2208 24616189

[41] Laine CM Wessman M Toiviainen-Salo S Kaunisto MA Mayranpaa MK Laine T. A novel splice mutation in PLS3 causes X-linked early onset low-turnover osteoporosis. J Bone Miner Res. (2015) 30:510–8. 10.1002/jbmr.2355 25209159

[42] Kampe AJ Costantini A Levy-Shraga Y Zeitlin L Roschger P Taylan F. PLS3 deletions lead to severe spinal osteoporosis and disturbed bone matrix mineralization. J Bone Miner Res. (2017) 32:2394–2404. 10.1002/jbmr.3233 28777485

[43] Kampe AJ Costantini A Makitie RE Jantti N Valta H Mayranpaa M. PLS3 sequencing in childhood-onset primary osteoporosis identifies two novel disease-causing variants. Osteoporos Int. (2017) 28:3023–32. 10.1007/s00198-017-4150-9 28748388 PMC5624974

[44] Tort O Tanco S Rocha C Bieche I Seixas C Bosc C. The cytosolic carboxypeptidases CCP2 and CCP3 catalyze posttranslational removal of acidic amino acids. Mol Biol Cell (2014) 25:3017–27. 10.1091/mbc.e14-06-1072 25103237 PMC4230590

[45] Sandoval N Platzer M Rosenthal A Dork T Bendix R Skawran B. Characterization of ATM gene mutations in 66 ataxia telangiectasia families. Hum Mol Genet. (1999) 8:69–79. 10.1093/hmg/8.1.69 9887333

[46] Palmio J Sandell S Hanna MG Mannikko R Penttila S Udd B. Predominantly myalgic phenotype caused by the c.3466G>A p.A1156T mutation in SCN4A gene. Neurology (2017) 88:1520–7. 10.1212/WNL.0000000000003846 28330959 PMC5395072

[47] Autiero M Waltenberger J Communi D Kranz A Moons L Lambrechts D. Role of PlGF in the intra- and intermolecular cross talk between the VEGF receptors Flt1 and Flk1. Nat Med. (2003) 9:936–43. 10.1038/nm884 12796773

[48] Estrada K Styrkarsdottir U Evangelou E Hsu YH Duncan EL Ntzani EE. Genome-wide meta-analysis identifies 56 bone mineral density loci and reveals 14 loci associated with risk of fracture. Nat Genet. (2012) 44:491–501. 10.1038/ng.2249 22504420 PMC3338864

[49] Peter M Couturier J Pacquement H Michon J Thomas G Magdelenat H. A new member of the ETS family fused to EWS in Ewing tumors. Oncogene (1997) 14:1159–64. 10.1038/sj.onc.1200933 9121764

[50] Tomlins S Rhodes D Perner S Dhanasekaran S Mehra R Sun X. Recurrent fusion of TMPRSS2 and ETS transcription factor genes in prostate cancer. Science (2005) 310:644–8. 10.1126/science.1117679 16254181

[51] Dupuis J Langenberg C Prokopenko I Saxena R Soranzo N Jackson AU. New genetic loci implicated in fasting glucose homeostasis and their impact on type 2 diabetes risk. Nat Genet. (2010) 42:105–16. 10.1038/ng.520 20081858 PMC3018764

[52] Kim JG Sun BH Dietrich MO Koch M Yao GQ Diano S. AgRP neurons regulate bone mass. Cell Rep. (2015) 13:8–14. 10.1016/j.celrep.2015.08.070 26411686 PMC5868421

[53] Vignaux G Ndong JD Perrien DS Elefteriou F. Inner ear vestibular signals regulate bone remodeling via the sympathetic nervous system. J Bone Miner Res. (2015) 30:1103–11. 10.1002/jbmr.2426 25491117 PMC4772960

