# Supplementary figures and images for: Rare Copy Number Variants in Array-Based Comparative Genomic Hybridization in Early-Onset Skeletal Fragility
Source: Front Endocrinol (Lausanne). 2018 Jul 10;9:380. doi: 10.3389/fendo.2018.00380 (PMC6048219; doi:10.3389/fendo.2018.00380)

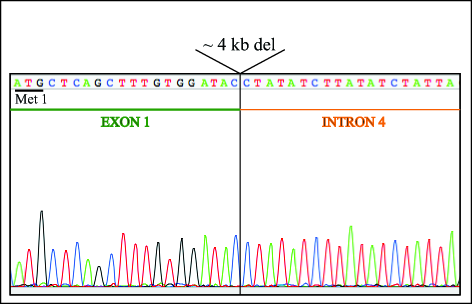

Supplement: Supplemental Figure S1 — Validation of the pathogenic finding in COL1A2 by breakpoint PCR followed by Sanger sequencing. The deletion starts in exon 1 and ends in intron 4. [file Image_1.TIF]

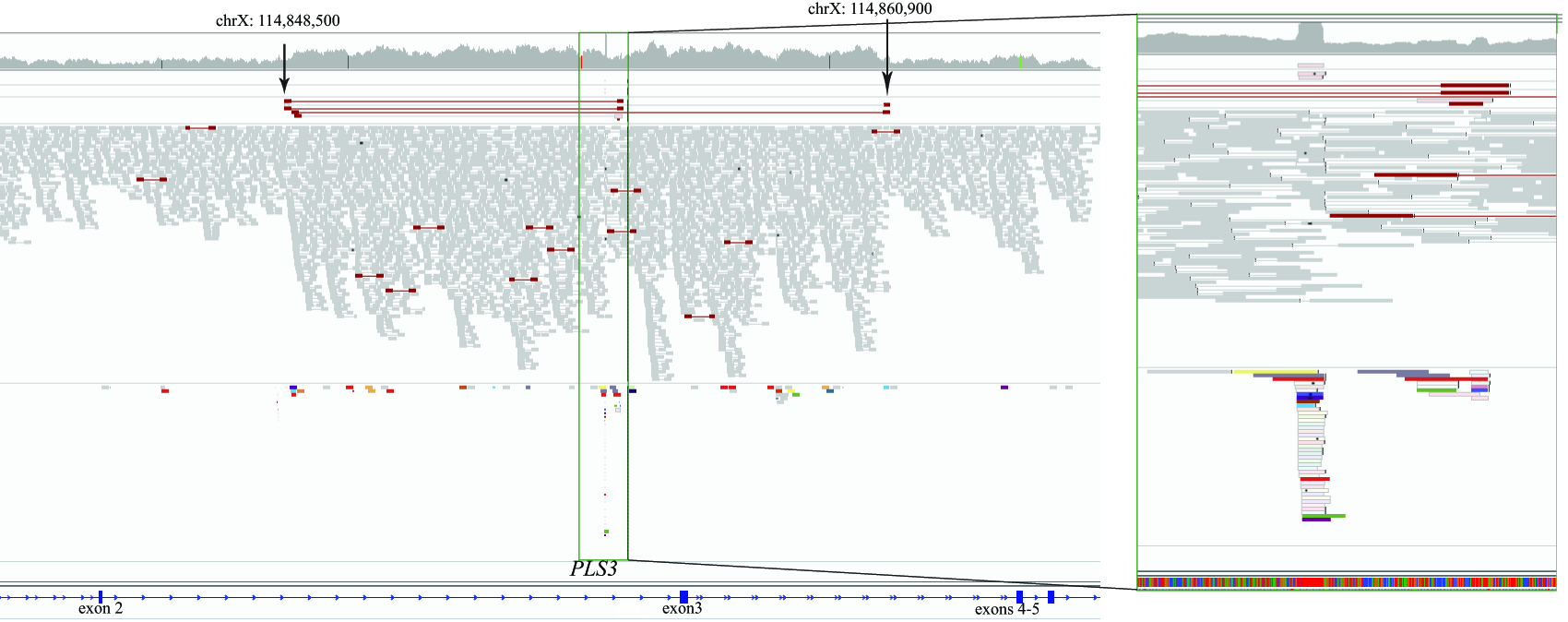

Supplement: Supplemental Figure S2 — Validation of the likely pathogenic finding in PLS3 by WGS. An increased coverage and a discordant orientation of some paired-end reads (red lines on the top of the figure) indicates the presence of a tandem duplication starting in intron 2 and ending in intron 3 of PLS3 (coordinates chrX: 114,848,500–114,860,900). On the right part of the figure a zoomed-in snapshot represents the area delimited by the green rectangle. The discordant pairs in this region are due to the misalignment of several reads (colorful reads) caused by the presence of a highly repetitive region (long red stretch on the bar below these reads). [file Image_2.TIF]
